# Sex differences in endothelial glycocalyx thickness and the response to glycocalyx‐targeted therapy among older adults

**DOI:** 10.14814/phy2.70428

**Published:** 2025-06-17

**Authors:** Colin J. Gimblet, Anthony J. Donato, Diana I. Jalal, Gary L. Pierce

**Affiliations:** ^1^ Department of Health and Human Physiology University of Iowa Iowa City Iowa USA; ^2^ Department of Internal Medicine University of Utah Salt Lake City Utah USA; ^3^ Iowa City VA Medical Center Iowa City Iowa USA; ^4^ Department of Internal Medicine University of Iowa Iowa City Iowa USA

**Keywords:** aging, glycocalyx, sex differences

## Abstract

Endothelial glycocalyx thickness declines with age, potentially increasing cardiovascular disease risk. However, sex differences in glycocalyx thickness and responses to glycocalyx‐targeted therapies remain unclear. This post hoc analysis examined sex differences in glycocalyx thickness and the effects of Endocalyx Pro supplementation in older adults. We analyzed data from 22 participants in a prior clinical trial (NCT06071728) that assessed 12‐week Endocalyx Pro (3712 mg/day) supplementation on vascular function. Glycocalyx thickness was estimated as the perfused boundary region (PBR) using the GlycoCheck, with higher PBR indicating smaller glycocalyx thickness. Postmenopausal females had higher PBR 4–25 than older males (2.11 ± 0.14 vs. 1.97 ± 0.13 μm; *p* = 0.02), particularly in microvessels 9–17 μm in diameter. Male sex (B [95% CI], −0.14 [−0.26, −0.02]; *p* = 0.02) and body mass index (BMI) (B [95% CI], −0.02 [−0.04, −0.01]; *p* = 0.01) were associated with lower PBR 4–25 in univariate analyses; however, when included in a multivariate model, the association with sex was attenuated (*p* = 0.15), while BMI remained significant (*p* = 0.04). After 12 weeks of Endocalyx Pro, PBR 4–25 increased in older males (+0.087 ± 0.148 μm) but decreased in postmenopausal females (−0.178 ± 0.148 μm; *p* = 0.009). In conclusion, we observed that postmenopausal females had smaller glycocalyx thickness, partially explained by BMI, and demonstrated a greater improvement with Endocalyx Pro, suggesting sex‐specific therapy effects.

## 1 INTRODUCTION

The endothelial glycocalyx is a dynamic extracellular layer atop the luminal surface of endothelial cells, functioning as a protective barrier between circulating blood and the vessel wall. The glycocalyx maintains endothelial function by preventing inflammatory cells from adhering to the endothelium, quenching oxidative stress via extracellular superoxide dismutase, and converting mechanical shear stress into nitric oxide (Foote et al., 2022). Notably, glycocalyx thickness declines with age (Machin et al., 2018), contributing to endothelial dysfunction and elevated cardiovascular risk in middle‐aged and older adults (Ikonomidis et al., 2021; Smilowitz et al., 2021). Therefore, preserving glycocalyx thickness represents a promising strategy for mitigating endothelial dysfunction and cardiovascular disease with aging.

Although advancing age increases cardiovascular disease risk in both sexes, females experience a more abrupt rise in risk during the menopausal transition. Premenopausal females generally have a lower cardiovascular disease risk than age‐matched males, but this protection is lost after menopause (Martin et al., 2025; Pérez‐López et al., 2009). One potential explanation is reduced glycocalyx thickness, as studies indicate that middle‐aged and older females have smaller glycocalyx thickness than their male counterparts (Ikonomidis et al., 2021; Triantafyllidi et al., 2020; Yuan et al., 2023). In this regard, interventions aimed at restoring glycocalyx thickness may represent a promising strategy to lower cardiovascular disease risk, particularly among postmenopausal females.

Endocalyx Pro is a patented glycocalyx‐targeted therapy formulated to support glycocalyx thickness in humans (Long & Vink, 2018). In a recent clinical trial, we found no significant improvement in glycocalyx thickness in older adults after 12 weeks of oral Endocalyx Pro supplementation (3712 mg per day) (Gimblet, Ernst, Bell, et al., 2024). Importantly, this primary analysis did not account for potential sex differences in glycocalyx structure or response to Endocalyx Pro. To address this gap, we conducted a post hoc analysis evaluating potential sex differences in glycocalyx thickness at baseline and the possible sex‐specific effects of Endocalyx Pro supplementation. We hypothesized that postmenopausal females would have smaller glycocalyx thickness than older males at baseline and a larger increase (i.e., improvement) in glycocalyx thickness following Endocalyx Pro supplementation.

## 2 METHODS

### 2.1 Study design

This post hoc analysis used data derived from a prior clinical trial (NCT06071728) that evaluated the impact of 12 weeks of oral Endocalyx Pro supplementation on vascular function in older adults. Comprehensive details of the study population, procedures, and clinical characteristics were previously published (Gimblet, Ernst, Bell, et al., 2024). Older males and postmenopausal females aged 60–85 years were recruited from Iowa City and the surrounding communities. Menopause status was assessed via questionnaire, and time since last menstrual cycle was self‐reported. Eligible participants were randomized to receive Endocalyx Pro (3712 mg/day) or a matching placebo for 12 weeks. Exclusion criteria included body mass index (BMI) ≥ 40 kg/m^2^, resting systolic blood pressure of ≥140 mmHg and/or diastolic blood pressure ≥ 100 mmHg, abnormal thyroid function, current tobacco use or tobacco use within the past 3 months, current use or use of hormone therapy within the past 6 months, history of diabetes mellitus, pulmonary/renal/hepatic disease, stroke, neurologic disorders, previous cardiovascular event (myocardial infarction, stent, bypass surgery, and heart failure), evidence of cardiovascular disease during a resting 12‐lead electrocardiogram, and allergy to artichokes, grapes, olives, or melons (Endocalyx Pro ingredients). The study procedures and informed consent documentation received approval from the University of Iowa Institutional Review Board and adhered to the ethical principles outlined in the Declaration of Helsinki. All participants signed written informed consent forms before taking part in the study.

### 2.2 Glycocalyx thickness

Glycocalyx thickness was quantified at baseline and 12 weeks by noninvasively imaging sublingual microvessels using a sidestream dark field imaging camera (KK Technology) and automated acquisition software (GlycoCheck, Microvascular Health Solutions, Alpine, UT) as previously described (Gimblet, Ernst, Bos, et al., 2024; Rovas et al., 2021). Five trials, each lasting 2–3 min, were conducted with at least ten 2‐s video recordings per trial, capturing 40 frames each. Values from all trials were averaged to obtain one measurement. Microvessels 4 to 25 μm in diameter were identified by contrasting red blood cells against the video background and segmented into 10 μm lengths. Recordings continued until 3000 segments were captured. The GlycoCheck software then estimated glycocalyx thickness by tracking the radial movement of red blood cells in each valid vessel segment and quantifying the perfused boundary region (PBR). This process involves determining the median width of red blood cell (RBCW) flow within the lumen and identifying the outer edge of the red blood cell‐perfused lumen (Dperf). PBR is then calculated using the formula: PBR = (Dperf − RBCW)/2, with a larger PBR value indicating a thinner glycocalyx.

### 2.3 Statistical analysis

Shapiro–Wilk tests were used to assess variables for normality. Normally distributed data are presented as mean ± standard deviation, non‐normally distributed data as median (interquartile range), and categorical data as number (percentage of participants). At baseline, unpaired Student's *t*‐tests were used to test differences in normally distributed variables, and unpaired Wilcoxon rank‐sum tests were used to test differences in non‐normally distributed variables between older males and postmenopausal females. At baseline, Pearson's correlation coefficient was used to evaluate the association between clinical characteristics and PBR 4–25, while linear modeling was used to assess associations of sex and BMI with PBR 4–25. Unpaired Student's *t*‐tests were applied to evaluate the change in PBR 4–25 stratified by treatment group and sex following supplementation. Significance was set at an alpha level of 0.05. All statistical analyses were performed in R, version 4.4.2 (R Foundation for Statistical Computing, Vienna, Austria).

## 3 RESULTS

### 3.1 Clinical characteristics

**FIGURE 1:**
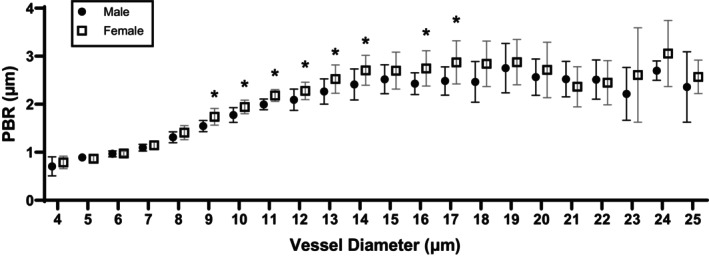
Glycocalyx thickness in males and post‐menopausal females, with higher PBR indicating lower glycocalyx thickness. Unpaired *t*‐tests were used to test differences in normally distributed variables and unpaired Wilcon rank‐sum tests were used to test differences in non‐normally distributed variables. PBR, perfused boundary region. *p < 0.05.

Baseline clinical characteristics between older males and postmenopausal females are shown in Table 1. The average duration of menopause among postmenopausal females was 15 ± 5 years. Older males and postmenopausal females were of similar age, mostly white, and the majority were not prescribed anti‐hypertensive medication. Additionally, older males and postmenopausal females had comparable systolic and diastolic blood pressure, circulating triglycerides, and low‐density lipoprotein cholesterol. However, older males had lower circulating total cholesterol and high‐density lipoprotein concentration than postmenopausal females. Moreover, older males had a higher BMI, although not statistically significant, and fasting blood glucose than postmenopausal females. Finally, PBR 4–25 was higher in postmenopausal females, and this appeared to be more pronounced in microvessels 9 to 14, 16, and 17 μm in diameter (Figure 1, Table S1).

**TABLE 1:** Baseline characteristics according to sex.

Variable	Males (*n* = 11)	Females (*n* = 11)	*p* Value
Age, years	66 (61,73)	63 (61,67)	0.37
Race, no. (%)			
White	10 (91)	11 (100)	0.99
Black	1 (9)	0 (0)	
Menopause duration, years	–	15 ± 5	–
Anti‐hypertensive med, no. (%)	4 (36.4)	2 (18.2)	0.63
Body mass index, kg/m^2^	26.8 ± 4.4	23.6 ± 2.7	0.05
Systolic BP, mmHg	126 ± 16	120 ± 12	0.32
Diastolic BP, mmHg	71 ± 11	68 ± 7	0.56
Glucose, mg/dL	91 (88,97)	84 (81,88)	0.02*
Total cholesterol, mg/dL	175 ± 32	212 ± 36	0.02*
Triglycerides, mg/dL	87 (51,122)	77 (66,86)	0.87
LDL, mg/dL	106 ± 24	128 ± 31	0.08
HDL, mg/dL	51 ± 19	67 ± 7	0.02*
PBR 4–25, μm	1.97 ± 0.13	2.11 ± 0.14	0.02*

*Note*: Variables are presented as mean ± standard deviation, median (interquartile range), or number (percentage of participants). Unpaired *t*‐tests were used to test differences in normally distributed variables and unpaired Wilcon rank‐sum tests were used to test differences in non‐normally distributed variables.

Abbreviations: BP, blood pressure; HDL‐C, high‐density lipoprotein; LDL, low‐density lipoprotein; PBR, perfused boundary region.

* *p*< 0.05.

### 3.2 Clinical characteristics associated with glycocalyx thickness at baseline

In the overall cohort (*n* = 22), age (*r* [95% CI], −0.27 [−0.62, 0.17]; *p* = 0.22), glucose (*r* [95% CI], −0.16 [−0.54, 0.28]; *p* = 0.48), total cholesterol (*r* [95% CI], 0.26 [−0.18, 0.61]; *p* = 0.25), triglycerides (*r* [95% CI], −0.13 [−0.52, 0.31]; *p* = 0.57), low‐density lipoprotein (*r* [95% CI], 0.26 [−0.18, 0.62]; *p* = 0.24), high‐density lipoprotein (*r* [95% CI], 0.22 [−0.22, 0.57]; *p* = 0.33), systolic blood pressure (*r* [95% CI], −0.35 [−0.67, 0.08]; *p* = 0.11), and diastolic blood pressure (*r* [95% CI], −0.34 [−0.67, 0.09]; *p* = 0.12) were not associated with PBR 4–25. In postmenopausal females, the duration of menopause was not correlated with PBR 4–25 (*r* [95% CI], 0.20 [−0.46, 0.72]; *p* = 0.56).

Table 2 highlights the associations of sex and BMI with PBR 4–25. In unadjusted linear models, PBR 4–25 was 0.14 μm lower in older males compared with postmenopausal females (B [95% CI], −0.14 [−0.26, −0.02]; *p* = 0.02). Additionally, each 1 kg/m^2^ increase in BMI was associated with a 0.02 μm reduction in PBR 4–25 (B [95% CI], −0.02 [−0.04, −0.01]; *p* = 0.01). In a multivariate model that included both sex and BMI, the association between sex and PBR 4–25 was attenuated (B [95% CI], −0.09 [−0.21, −0.04]; *p* = 0.15), whereas the relation between BMI and PBR 4–25 persisted (B [95% CI], −0.02 [−0.03, 0.00]; *p* = 0.04).

**TABLE 2:** Association of sex and body mass index with glycocalyx thickness.

Variable	Model 1		Model 2		Model 3	
	B (95% CI)	*p* Value	B (95% CI)	*p* Value	B (95% CI)	*p* Value
Male sex	−0.14 (−0.26, −0.02)	0.02*	–	–	−0.09 (−0.21,0.04)	0.15
BMI, kg/m^2^	–	–	−0.02 (−0.04, −0.01)	0.01*	−0.02 (−0.03, −0.01)	0.04*

*Note*: PBR 4–25 was used to estimate glycocalyx thickness, with higher PBR indicating lower glycocalyx thickness. A generalized linear model was used to assess the relation between sex, body mass index, and perfused boundary region. Model 1: Male sex with PBR 4–25. Model 2: BMI with PBR 4–25. Model 3: Male sex and BMI with PBR 4–25.

Abbreviations: BMI, body mass index; PBR, perfused boundary region.

* *p*< 0.05.

### 3.3 Sex‐specific effects of Endocalyx pro on glycocalyx thickness

**FIGURE 2:**
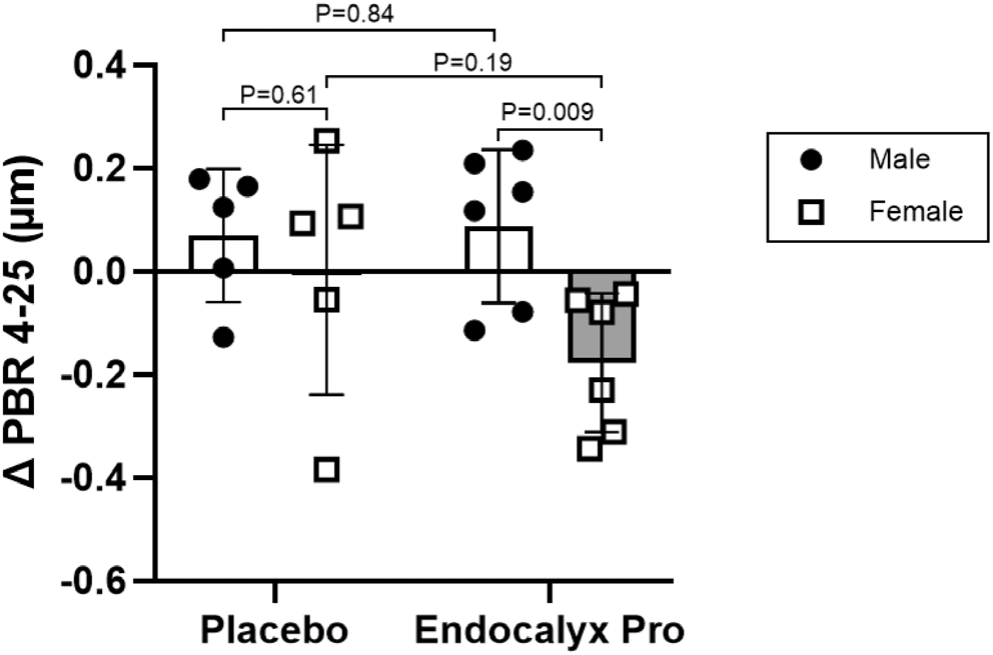
Change in glycocalyx thickness after 12 weeks of supplementation stratified by treatment group and sex. Unpaired Student's *t*‐tests were applied to evaluate the change in PBR 4‐25. PBR, perfused boundary region.

Figure 2 depicts the changes in glycocalyx thickness stratified by treatment group and sex following 12‐week oral supplementation with Endocalyx Pro. In the placebo group, the mean change in PBR 4–25 was 0.069 ± 0.129 μm in older males compared with 0.002 ± 0.242 μm in postmenopausal females, with a nonsignificant between‐group mean difference (*p* = 0.61). Conversely, in the Endocalyx Pro group, older males exhibited a mean increase in PBR 4–25 of 0.087 ± 0.148 μm, while postmenopausal females demonstrated a reduction in PBR 4–25 of −0.178 ± 0.148 μm, yielding a significant between‐group mean difference (*p* = 0.009). There were no significant differences in PBR 4–25 between placebo and supplement groups in either sex (*p* > 0.05).

## 4 DISCUSSION

This post hoc analysis evaluated sex differences in glycocalyx thickness and the response to oral Endocalyx Pro supplementation among older adults. We observed that postmenopausal females had smaller glycocalyx thickness than older males, particularly in vessels 9 to 17 μm in diameter. Additionally, postmenopausal females demonstrated a greater increase in glycocalyx thickness following 12 weeks of Endocalyx Pro supplementation. These findings suggest that smaller glycocalyx thickness in postmenopausal females compared with age‐matched males may influence the efficacy of glycocalyx‐targeted therapies in this population.

Biological sex and hormonal status are important determinants of cardiovascular disease risk, particularly in females. The rise in cardiovascular disease risk following menopause is largely attributed to altered sex hormone production (Boese et al., 2017). Despite this, few studies have investigated the effect of sex hormones on glycocalyx thickness with aging. Previous studies have demonstrated that middle‐aged and older females have smaller glycocalyx thickness compared with males (Ikonomidis et al., 2021; Triantafyllidi et al., 2020; Yuan et al., 2023). However, to our knowledge, this is the first study to directly evaluate biological sex differences in glycocalyx thickness among older males and postmenopausal females. Our results confirm that postmenopausal females had smaller glycocalyx thickness compared with older males, though this association was attenuated when adjusted for BMI. Additionally, oral Endocalyx Pro supplementation resulted in a greater increase in glycocalyx thickness among postmenopausal females, raising the possibility that sex hormones modulate glycocalyx thickness with aging. However, we did not observe significant differences in glycocalyx thickness between the placebo and Endocalyx Pro groups in postmenopausal females, which may be attributed to high variability in PBR 4–25 changes among those receiving placebo. These findings underscore the need for larger, adequately powered studies to clarify the sex‐specific effects of glycocalyx‐targeted therapies.

Preclinical data support the role of sex hormones in glycocalyx regulation. Estrogen treatment protects against glycocalyx degradation in human umbilical vein endothelial cells subjected to biomimetic shock conditions (Diebel et al., 2021). Moreover, the concentration of circulating glycocalyx components varies throughout the menstrual cycle in non‐pregnant females (Hulde et al., 2018). Specifically, the glycocalyx components syndecan‐1 and heparan sulfate are elevated when females transition out of ovulation and into the luteal phase (Hulde et al., 2018). Of note, serum progesterone, but not estrogen, was lower during ovulation and elevated in the luteal phase (Hulde et al., 2018), suggesting that estrogen may have a protective effect on the glycocalyx while progesterone may have opposing effects. Nevertheless, no longitudinal studies have evaluated the impact of estrogen and progesterone on glycocalyx thickness over time. Thus, further research is warranted to clarify the direct role of estrogen and progesterone on glycocalyx degradation in postmenopausal females.

Although sex hormones provide a plausible mechanism for the observed sex differences in glycocalyx thickness, other factors may also contribute. In our study, BMI was the only clinical characteristic that differed between older males and postmenopausal females and was associated with glycocalyx thickness, with greater BMI linked to larger glycocalyx thickness. This aligns with preclinical data revealing that higher body mass was associated with larger glycocalyx thickness in genetically diverse mice (Zheng et al., 2022). Conversely, higher BMI was correlated with smaller glycocalyx thickness in microvessels 5–9 μm in diameter in hypertensive adults with a greater cardiovascular disease risk profile than adults in our study (Triantafyllidi et al., 2018). Given these inconsistencies, additional research is needed to determine whether BMI contributes to sex differences in glycocalyx thickness independent of hormonal changes.

Several limitations should be considered. First, this was a post hoc analysis with a limited sample size and not powered to detect significant sex differences in endothelial glycocalyx thickness. Therefore, our findings should be interpreted as exploratory and used to guide the design of future, adequately powered trials. Larger studies are essential to confirm whether sex‐specific differences influence the efficacy of glycocalyx‐targeted therapies in older adults. Additionally, our study was not adequately powered to assess whether antihypertensive medication use influences the sex‐specific response to Endocalyx Pro, which remains an important area for future research. Next, we did not measure circulating biomarkers of endothelial glycocalyx shedding, such as syndecan‐1, heparan sulfate, and hyaluronan. Although elevated levels of these biomarkers have been observed in several disease states, they show only a moderate correlation with the structural integrity of the endothelial glycocalyx assessed via the GlycoCheck (Hahn et al., 2021). Nonetheless, including these circulating biomarkers would have enhanced our study by providing complementary information on their relation with PBR in older adults and potential sex differences between males and postmenopausal females. Finally, the potential confounding effect of BMI on glycocalyx thickness warrants further investigation. Although postmenopausal females in our study had lower BMI, body composition measures such as fat mass and fat‐free mass were not assessed, and our analysis was not powered to fully evaluate BMI as a confounder. Future trials should consider adjusting for sex, BMI, and body composition, or including stratified analyses to isolate their independent effects on glycocalyx thickness.

In conclusion, we observed that postmenopausal females exhibit smaller glycocalyx thickness than older males, potentially because of differences in BMI. Additionally, Endocalyx Pro may have sex‐specific effects on glycocalyx thickness in older adults. Larger, adequately powered studies are needed to validate these findings and further explore the role of sex hormones and BMI in glycocalyx regulation.

## FUNDING INFORMATION

This study was supported by the American Heart Association predoctoral Fellowship Grant (23PRE1012593). Colin J. Gimblet is supported by the Diabetes Research Center Training Grant (NIH T32DK112751). Gary L. Pierce is supported by the Russell B. Day and Florence D. Day Endowed Chair in Liberal Arts and Sciences at the University of Iowa.

## ETHICS STATEMENT

The study procedures and informed consent documentation received approval from the University of Iowa Institutional Review Board and adhered to the ethical principles outlined in the Declaration of Helsinki. All participants signed written informed consent forms before taking part in the study.

## DISCLAIMER

Microvascular Health Solutions provided Endocalyx Pro and the matching placebo free of charge but were not involved in data collection, analysis, or interpretation of the results.

## Supporting information


Table S1.
