# Supplementary material for: Sex differences in endothelial glycocalyx thickness and the response to glycocalyx‐targeted therapy among older adults
Source: Physiol Rep. 2025 Jun 17;13(12):e70428. doi: 10.14814/phy2.70428 (PMC12172339; doi:10.14814/phy2.70428)
Supplement: Supplementary file 1 — Table S1. [file PHY2-13-e70428-s001.docx]

| **Supplemental Table 1.** PBR in males and post-menopausal females according to vessel diameter. | | | | |
| --- | --- | --- | --- | --- |
| Vessel diameter | Males (n=11)  PBR µm | Females (n=11)  PBR µm | Difference (95% CI) | P Value |
| 4 µm | 0.71±0.20 | 0.79±0.13 | 0.08 (-0.08,0.24)^a^ | 0.29 |
| 5 µm | 0.89±0.07 | 0.87±0.06 | -0.02 (-0.09,0.04)^a^ | 0.408 |
| 6 µm | 0.97±0.06 | 0.97±0.05 | 0.01 (-0.04,0.06)^a^ | 0.749 |
| 7 µm | 1.10±0.07 | 1.14±0.09 | 0.05 (-0.02,0.12)^a^ | 0.172 |
| 8 µm | 1.31±0.11 | 1.41±0.15 | 0.10 (-0.02,0.21)^a^ | 0.100 |
| 9 µm | 1.54±0.12 | 1.74±0.17 | 0.19 (0.06,0.33)^a^ | 0.006* |
| 10 µm | 1.78±0.15 | 1.94±0.14 | 0.16 (0.04,0.30)^a^ | 0.015* |
| 11 µm | 2.00±0.11 | 2.18±0.12 | 0.19 (0.08,0.29)^a^ | 0.001* |
| 12 µm | 2.09±0.22 | 2.28±0.18 | 0.18 (0.00,0.36)^a^ | 0.045* |
| 13 µm | 2.27±0.26 | 2.52±0.30 | 0.26 (0.01,0.51)^a^ | 0.043* |
| 14 µm | 2.41±0.32 | 2.71±0.31 | 0.29 (0.01,0.58)^a^ | 0.042* |
| 15 µm | 2.52±0.30 | 2.70±0.38 | 0.18 (-0.13,0.49)^a^ | 0.236 |
| 16 µm | 2.43±0.23 | 2.75±0.37 | 0.32 (0.04,0.59)^a^ | 0.025* |
| 17 µm | 2.48±0.30 | 2.87±0.45 | 0.39 (0.05,0.73)^a^ | 0.026* |
| 18 µm | 2.25 (2.18,2.68) | 3.05 (2.34,3.24) | 0.80 (-0.27,1.03)^b^ | 0.08 |
| 19 µm | 2.75±0.51 | 2.88±0.47 | 0.13 (-0.31,0.56)^a^ | 0.555 |
| 20 µm | 2.56±0.38 | 2.71±0.58 | 0.15 (-0.31,0.61)^a^ | 0.486 |
| 21 µm | 2.52±0.37 | 2.36±0.42 | -0.16 (-0.51,0.19)^a^ | 0.355 |
| 22 µm | 2.51±0.41 | 2.45±0.46 | -0.06 (-0.48,0.36)^a^ | 0.752 |
| 23 µm | 2.14 (1.82,2.59) | 2.41 (2.08,2.54) | 0.27 (-0.44,0.74)^b^ | 0.44 |
| 24 µm | 2.70±0.20 | 3.06±0.69 | 0.36 (-0.22,0.94)^a^ | 0.211 |
| 25 µm | 2.36±0.73 | 2.57±0.35 | 0.21 (-0.39,0.81)^a^ | 0.470 |
| Higher PBR indicates lower glycocalyx thickness. Unpaired t-tests were used to test differences in normally distributed variables and unpaired Wilcon rank-sum tests were used to test differences in non-normally distributed variables. The mean or median difference was calculated by subtracting the value in males from the value in females, such that a positive value indicates a higher value in females compared to males. PBR, perfused boundary region. * P < 0.05  ^a^ Mean difference  ^b^ Median difference | | | | |
